# Gravitational lensing: a unique probe of dark matter and dark energy

**DOI:** 10.1098/rsta.2009.0209

**Published:** 2010-03-13

**Authors:** Richard S. Ellis

**Affiliations:** Astronomy Department, MS 249-17, California Institute of Technology, Pasadena, CA 91125, USA

**Keywords:** gravitational lensing, observational cosmology, large-scale structure, galaxy evolution

## Abstract

I review the development of gravitational lensing as a powerful tool of the observational cosmologist. After the historic eclipse expedition organized by Arthur Eddington and Frank Dyson, the subject lay observationally dormant for 60 years. However, subsequent progress has been astonishingly rapid, especially in the past decade, so that gravitational lensing now holds the key to unravelling the two most profound mysteries of our Universe—the nature and distribution of dark matter, and the origin of the puzzling cosmic acceleration first identified in the late 1990s. In this non-specialist review, I focus on the unusual history and achievements of gravitational lensing and its future observational prospects.

## 1. Introduction

In selecting a topic to write about in celebration of the Royal Society’s 350th anniversary, I thought it appropriate to write a non-specialist review of the progress that has been made in recent years in utilizing the phenomenon of *gravitational lensing*—the deflection of light by gravitating mass. This seems particularly appropriate because, at the time of writing, astronomers are celebrating, as part of the International Year of Astronomy, the ninetieth anniversary of the famous 1919 solar eclipse expeditions organized by Arthur Eddington and Frank Dyson that first demonstrated the deflection of starlight predicted by Einstein’s theory of general relativity. Although Einstein and Eddington were sceptical of the long-term observational utility of gravitational lensing, a renaissance began in the 1970s following the delivery of improved astronomical detectors and large telescopes. The subject has grown apace since the launch of the *Hubble Space Telescope*, such that gravitational lensing is now one of the most powerful tools in the armoury of the modern astronomer, contributing significantly to determining the growth of the large-scale structure of the Universe and the evolution of galaxies within it.

Via this short review, I hope to catalogue this progress, illustrating some of the recent highlights as well as discussing how gravitational lensing is likely to play a key role in making progress in determining the distribution of dark matter and possibly even its nature, as well as exploring the implications of the ‘dark energy’ proposed to explain the cosmic acceleration discovered in the late 1990s.

## 2. Early history

### (a) Classical calculations

There are many excellent reviews of gravitational lensing (e.g. Blandford & Narayan 1992; Schneider *et al.* 2006 and references therein), several of which cover the early history. The earliest known mention of light being deflected by massive objects is the first query contained in Newton's *Opticks* in 1704 (page 132):

Do not Bodies act upon Light at a distance, and by their action bend its rays; and is not this action strongest at the least distance?

Newton’s queries were generally posed as rhetorical questions. Unfortunately, the query does not make a distinction between gravitational light bending, i.e. the action of gravity on a corpuscle, and more conventional optical phenomena. Although it was left to later workers to calculate the gravitational deflection caused by the Sun on starlight, Newton had already made similar calculations for a medium with varying density in order to calculate the effects of refraction in the Earth’s atmosphere. As a result, in 1784, John Mitchell was able to use Newton’s *Opticks* to argue that light would be weakened (redshifted) in climbing out of a gravitational well.

Henry Cavendish in 1784 is credited with the first (unpublished) calculation of the deflection angle *δ* of a corpuscular light ray following a hyperbolic trajectory and the origin of the (Newtonian) equation *δ*=2*GM*/*Rc*^2^. Subsequently, von Soldner (1804) published a similar calculation deriving a deflection of 0.84 arcsec for stars viewed close to the limb of the Sun. Von Soldner additionally discussed the practicality of verifying this prediction, but his work, as well as that of Cavendish, was largely forgotten as the corpuscular theory of radiation was increasingly discredited in favour of wave theories of light. Not only was there confusion as to whether a deflection was expected for a light wave, but the small value predicted by von Soldner was also considered unobservable.

### (b) Einstein and the solar deflection

In 1911, Einstein calculated a relativistic version of the solar deflection and derived a similar result to that achieved by von Soldner a hundred years earlier, 0.875 arcsec. The similarity in the conclusion led some (Lenard 1918) to accuse Einstein of plagiarism, but the physical principles behind the two calculations are quite different. In the classical calculation, it is assumed that light can be accelerated and decelerated like a normal mass particle, whereas in Einstein’s calculation the deflection is based on gravitational time dilation. In 1915, Einstein considered the additional deflection arising from the curvature of space around the Sun in his newly published general theory, from which he derived *δ*=4*GM*/*Rc*^2^ and a solar deflection of 1.75 arcsec.

Beginning in 1912, Einstein sought out observers who could verify his predicted deflection (notwithstanding that his prediction doubled in value in the next 7 years!). He corresponded with George Ellery Hale at the Carnegie Observatories regarding the possibility of observing the much smaller deflection around the planet Jupiter, eventually concluding that photographs taken at a total solar eclipse were the only realistic option.

The observational race to prove or disprove Einstein’s theory is a fascinating story well documented in several recent books (Coles 1999; Crelinsten 2006; Stanley 2007; Gates 2009) and television documentaries. Einstein’s chosen astronomer, Erwin Findlay-Freundlich, failed on numerous occasions, most spectacularly when he was arrested as a German national in the Crimea at the August 1914 eclipse, war being declared that very month! William Wallace Campbell, Director of the Lick Observatory, was likewise motivated to test Einstein’s theory (although perhaps more sceptically than Findlay-Freundlich). He was also unfortunate in 1914; as a US citizen he was free to leave Russia but his equipment was impounded. At a subsequent eclipse in Washington State in 1918, Campbell had to make do with inferior equipment and eventually concluded that there was no deflection. He was poised to publish his rejection of Einstein’s theory when he heard of Eddington’s likely verification during a visit to London in 1919. In a famous telegram, he urged his Californian colleagues to hold off submitting the paper.

### (c) The 1919 eclipse

The Astronomer Royal, Frank Dyson, first proposed the 29 May 1919 eclipse expedition, noting that the Sun would be in the rich field of the Hyades star cluster—a rare opportunity! Eddington had played a key role in promoting Einstein’s theory and took the lead in the organization. Eddington and his assistant Cottingham visited the island of Príncipe off the coast of West Africa (now part of the Democratic Republic of Sao Tomé and Príncipe); another team (Crommelin and Davidson) visited Sobral, Brazil. The results, confirming the full deflection predicted by general relativity, were presented in November 1919 (Dyson *et al.* 1920).

Some have argued that Eddington was so blinded by his enthusiasm for Einstein’s theory that he was biased in his analysis of the Príncipe and Sobral plates, discarding discrepant data (Waller 2002). At Príncipe, only two plates were successfully exposed with an astrograph, giving a mean deflection of 1.61±0.30 arcsec. At Sobral, more plates were taken with a similar astrograph, giving a smaller deflection of approximately 0.93 arcsec. Use of a second telescope at Sobral gave a deflection of 1.90±0.11 arcsec. It has been argued that Eddington dispensed unnecessarily with the discrepant Sobral astrograph results to force agreement with Einstein.

A recent re-analysis (Kennefick in press) shows that this was not the case. The Sobral astrograph plates were out of focus as a result of the rapid change in temperature during totality. This meant that it proved very difficult to establish a proper plate scale. In fact, it was Dyson who discarded these results. His subsequent, more careful, analysis of these plates after publication gave a deflection of 1.52 arcsec. In 1979, the Sobral plates were more accurately re-measured with a plate-measuring machine, yielding a deflection of 1.55±0.32 arcsec (Harvey 1979).

**Figure 1.**
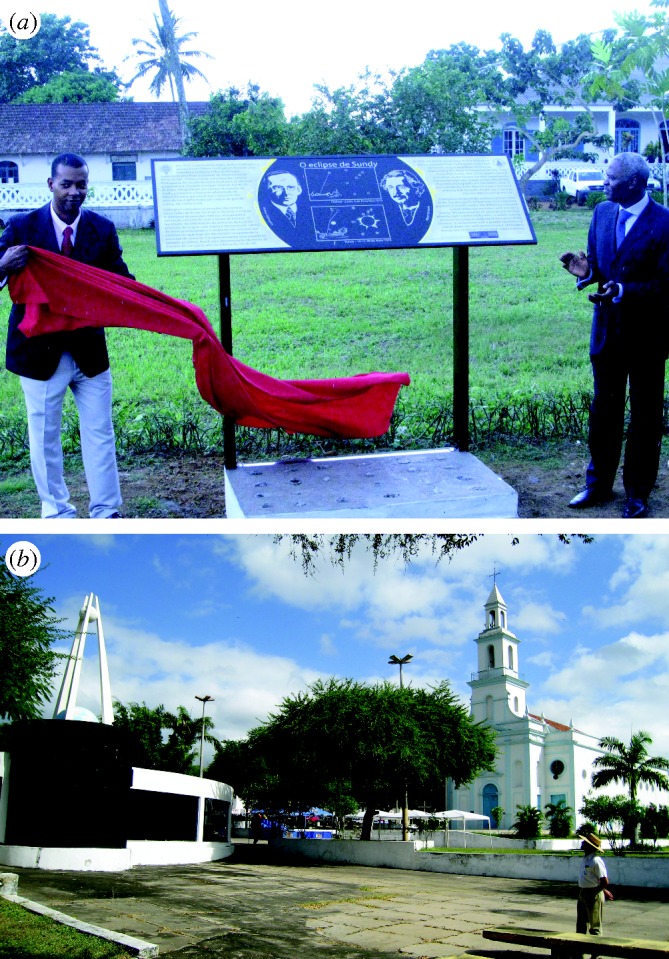
(*a*) Unveiling a new commemorative plaque to Eddington and Einstein at the Roça Sundy site in Príncipe (photo courtesy of Richard Massey). (*b*) The museum commemorating the 1919 eclipse at the relevant site in Sobral, Brazil, with its current Director, Dr F. de Almeida.

To mark the 2009 International Year of Astronomy, my colleagues and I recently visited both historic sites. With funding from the IAU and Royal Astronomical Society, a new commemorative plaque (figure 1*a*) was placed at the site in Príncipe (Ellis *et al.* 2009). An eclipse museum has been in place for 10 years at Sobral (figure 1*b*).

Given that general relativity had already demonstrated some measure of success in predicting the perihelion precession of the planet Mercury (Einstein 1916), it is interesting to ask why it is that the solar deflection is regarded as the key observation that catapulted Einstein to international fame. One possible reason is simply the fact that the concept of gravitational lensing—a term possibly introduced by Eddington himself—captured the imagination of the public as an indication of a new era in science heralded by the young Einstein. As an indication of the popular appeal of the experiment, a (now lost) cartoon referred to by some early reviewers apparently depicts Sherlock Holmes spying a criminal behind a wall, the light bent around the corner; the caption claims ‘Gravitational, my dear Watson!’

### (d) A lean 60 years: 1919–1979

Eddington and Einstein were curiously reticent about possible applications of gravitational lensing. Chwolson (1924) illustrated how lensing can produce multiple images of a distant source (a phenomenon now termed *strong lensing*) but, as its occurrence depends on the precise alignment of a source and deflector, it was reasonable to conclude that the probability of observing such phenomena would be very small. As a good illustration of thinking at the time, Einstein (1936), urged by Mandl, discussed what Paczynski later called *microlensing*—the temporary brightening of a star due to the magnification induced by a foreground object that crosses the line of sight to the observer. In this rare post-1919 article about lensing by its discoverer, he states ‘of course there is no hope of observing this phenomenon’.

The Caltech astronomer Fritz Zwicky emerges as a lone prophet from this era. In a brief article seemingly neglected at the time (Zwicky 1937), he opines that galaxies and galaxy clusters would be far more useful lenses and, with great vision, imagines that lensing via such systems would enable detailed studies of otherwise too faint distant systems as well as providing constraints on the total (dark plus visible) masses of the lenses. In the 1960s, Barnothy & Barnothy (1968) became tireless advocates of Zwicky’s position. The mathematics of multiply imaged geometries was further developed independently by Klimov (1963), Liebes (1964) and Refsdal (1964*a*). Refsdal (1964*b*) demonstrated that, if a background lensed source such as a quasar is variable in its light output, an absolute distance scale can be determined by measuring the time delay in the arrival of light observed in its multiple images; this offers a geometric route to measuring the rate of expansion of the Universe.

### (e) The renaissance

Why did it take until 1979 before further observational progress was made in gravitational lensing? Zwicky was correct that galaxies and galaxy clusters serve as more probable lenses than individual stars, but even so three factors seriously limit the visibility of lensed images.

Firstly, it is useful to introduce the concept of *optical depth* τ in considering the probability of an alignment. The optical depth that a particular class of galaxy *g* provides in forming multiple images is approximately equal to the total mass density of that population as a fraction of the total energy density of the Universe, *τ*≈*Ω*_*g*_. Since the mass density of galaxies is *Ω*_*g*_∼10^−3^, it follows that many thousand foreground galaxies must be surveyed to find a suitable configuration; strong lensing by galaxies is a rare phenomenon!

**Figure 2.**
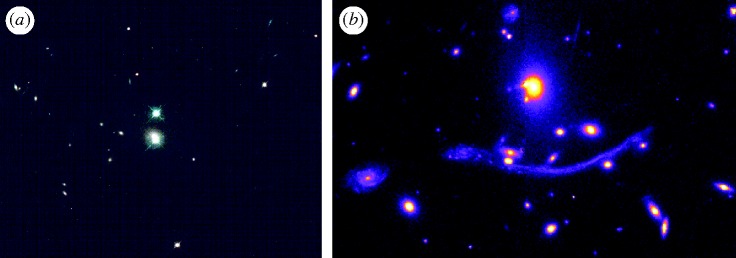
(*a*) *Hubble Space Telescope* image of the first gravitationally lensed source, the high-redshift quasar SBS 0957 + 561. The two nearly comparable images of the same background source are produced by the lensing effect of a foreground elliptical galaxy. (*b*) The remarkable ‘giant arc’ in the rich cluster of galaxies, Abell 370. The image is that of a distant galaxy distorted by the gravitational potential of the foreground cluster.

Secondly, as in conventional optics, the background source must be substantially more distant than the lens. The optimum configuration has the observer–lens–source equidistant in relativistic units. Until the 1960s, very few high-redshift sources were known. Only as quasar surveys yielded many distant sources in the 1970s did it finally become likely that one would be found behind a foreground galaxy. The first example, SBS 0957+561 A/B, was verified spectroscopically by Walsh *et al.* (1979) to represent two images of the same distant (redshift *z*=1.413) quasar. The lensing galaxy has a redshift *z*=0.355 (figure 2*a*).

The third limiting factor in locating lensed images arises from the fact that surface brightness is conserved in the lensing process (as it is in conventional optics). However, as surface brightness dims with increased redshift *z* as (1+*z*)^4^ due to relativistic effects associated with the expansion of the Universe, many lensed images viewed through galaxy clusters were simply too faint to be detected and lay undiscovered until the 1980s when charge-coupled devices became common on large ground-based telescopes. The increased sensitivity led to the discovery in the mid-1980s of giant arcs such as that viewed in the cluster Abell 370 (*z*=0.37, figure 2*b*). For a few years, there was some speculation as to the origin of these strange features. Eventually, Soucail *et al.* (1988) confirmed, with a spectrum, that the arc in Abell 370 is the distorted image of a single background galaxy at redshift *z*=0.724.

## 3. Scientific highlights illustrating the variety of lensing phenomena

The earlier cited reviews give a useful pedagogical introduction to the physics of gravitational lensing, including how, for example, multiple images are formed. Rather than reproduce the mathematics, I will attempt to illustrate the three basic modes of lensing via some recent scientific highlights.

### (a) Strong lensing

**Figure 3.**
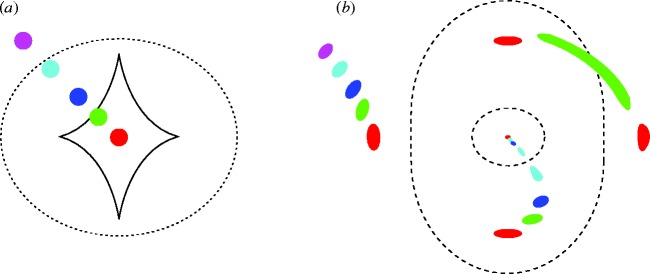
Deployment of multiple images for a strong elliptical lens. (*a*) Source-plane view, with various locations of the source indicated by coloured dots. Lines represent lensing caustics. (*b*) Image-plane view (what the observer sees); dotted lines represent critical lines for the relevant source distance. The variously coloured multiple images correspond to the changing position of the source. When the source approaches the caustic, the number of multiple images increases. As surface brightness is conserved, the amount of distortion in the image represents a magnification in the total received brightness as well as a spatial enlargement. Both features are useful advantages of lensing in the study of distant galaxies.

In the 1919 solar eclipse, starlight was only marginally deflected by the Sun’s gravitational field. However, for an optimal arrangement, a lens whose mass density in projection is above a critical value can multiply image and magnify a background source. This is known as strong lensing (for an up-to-date review, see Treu in press). The strong lensing phenomenon can be viewed in terms of an optical mapping between a (true) *source plane* and an observed *image plane*. Lensing differs from conventional optics in that there is no single focal point but rather lines of (theoretically) infinite magnification called *critical lines*. Transferred to the source plane, these lines become *caustics*. The location of these lines depends on the relative distances of the source and lens and, of course, the distribution of matter in the lens. The position of the background source with respect to the caustic appropriate for its distance governs the arrangement of the multiple images and the image magnifications (figure 3). I have selected two applications based on the phenomenon of strong lensing that illustrate recent progress in galaxy formation and cosmology.

#### (i) The distribution of dark matter in elliptical galaxies

**Figure 4.**
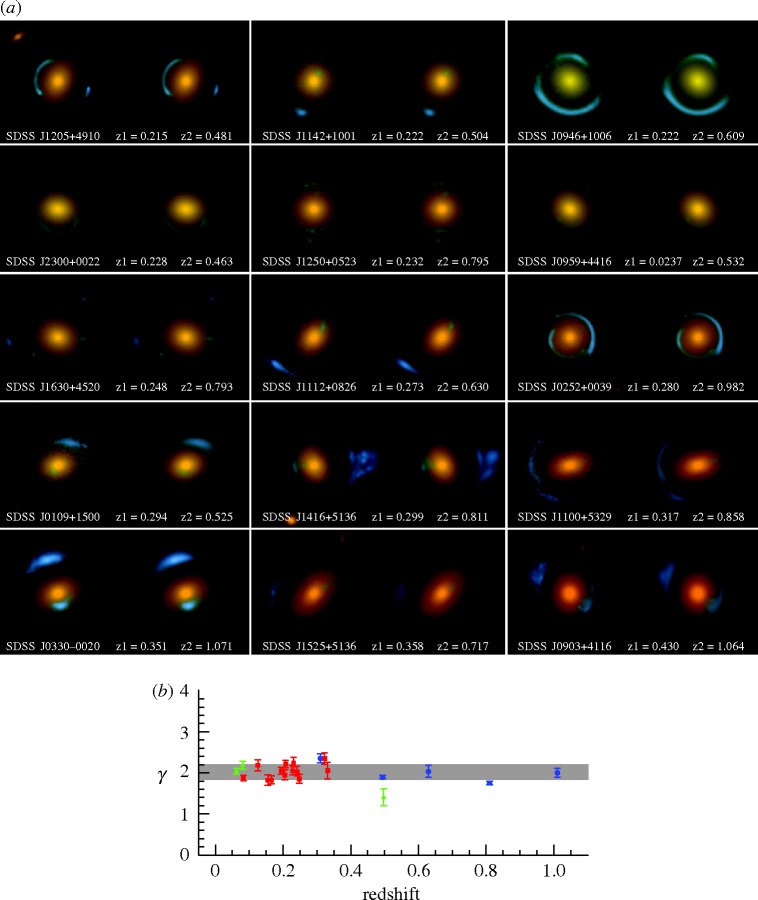
(*a*) *Hubble Space Telescope* images of a selection of elliptical lenses from the SLACS survey (Bolton *et al.* 2008). The blue ring-like features represent the distorted (and magnified) images of background galaxies lensed by the foreground elliptical galaxy (orange). (*b*) Distribution of the logarithmic slope *γ* of the mass density distribution 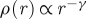 derived from a combination of the lensing geometry and stellar dynamics of the lensing elliptical. The remarkable uniformity in the mass profile argues for early formation concurrent with the assembly of a massive dark matter halo.

The notion that galaxies are surrounded by haloes of dark matter had become commonplace by the early 1980s. But how can we quantify the distribution of dark matter around galaxies and verify its role in galaxy formation, given that it is invisible? Elliptical galaxies are compact and dense and thus serve as excellent gravitational lenses. Using spectroscopic data from the Sloan Digital Sky Survey, the SLACS^1^ team has so far isolated 98 ellipticals that strongly lens background blue star-forming galaxies at moderately high redshift (Bolton *et al.* 2008). Since the redshifts of both the lens and the background source are known, the lensing geometry, revealed by *Hubble Space Telescope* images (figure 4*a*), defines the total mass interior to the critical line (or ‘Einstein radius’) irrespective of whether that material is shining. Together with a dynamically based mass on a smaller physical scale derived from the dispersion of stellar velocities in the lensing galaxy itself, the total mass density in the lens as a function of galactocentric distance *ρ*(*r*) can be determined. Across a wide range in cosmic time and lens mass, the total mass distribution is remarkably uniform, following an isothermal distribution, 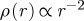 (figure 4*b*). This distribution is spatially more extended than that of the visible baryons, demonstrating clearly the existence of dark matter. Finally, the total mass distribution appears to share the ellipticity and orientation of the light (Koopmans *et al.* 2006). These important results confirm that the early formation of massive dark matter haloes played the key role in encouraging a rapid formation of the cores of massive galaxies.

#### (ii) Locating and studying the magnified images of distant galaxies

**Figure 5.**
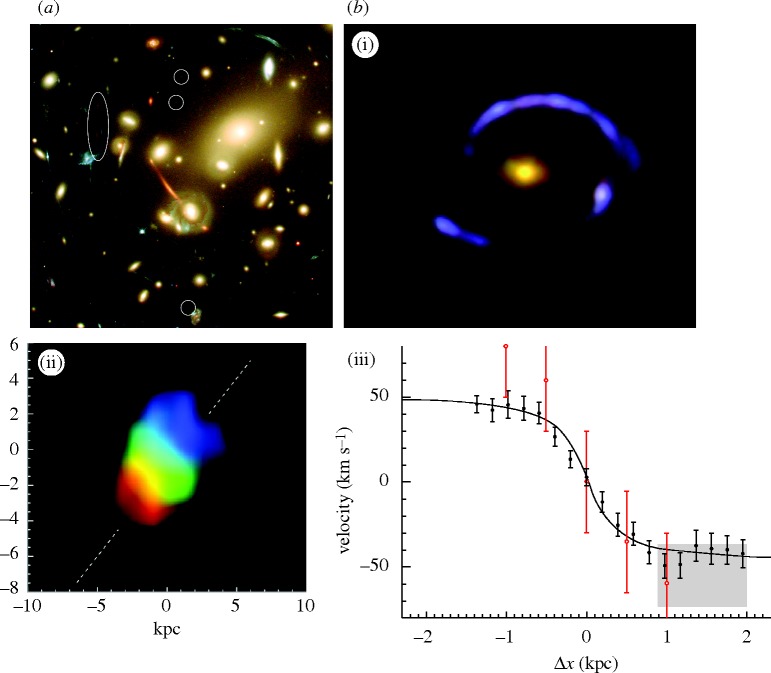
(*a*) *Hubble Space Telescope* image of the rich cluster Abell 2218 showing a plethora of distorted arcs and multiple images. Along the critical lines of high magnification were found two of the highest-redshift magnified sources known (at the time of discovery). One is a close pair of images representing a source at redshift 5.7 (Ellis *et al.* 2001); the other is a triply imaged system at redshift 6.8 (Kneib *et al.* 2004). (*b*) A highly magnified star-forming galaxy at *z*=3.1 for which resolved spectroscopy reveals a rotating disc (Stark *et al.* 2008): (i) actual image with the *Hubble Space Telescope*, (ii) reconstructed source-plane image with colour-coded velocity field, and (iii) velocity versus major axis position.

In his remarkably prescient article, Zwicky (1937) suggested that clusters of galaxies could be used as ‘natural telescopes’ to search for magnified images of very distant galaxies, thereby extending the reach of our existing telescopes. In the past 5 years, this has become a very effective way to locate and understand the properties of the earliest galaxies seen when the Universe was only 10–15% of its current age. A rich cluster of galaxies presents a much larger cross section to the background population than a single galaxy, and so the likelihood of magnified images is much greater; indeed, many clusters reveal a plethora of multiple images (figure 5*a*). On the other hand, the distribution of mass in a cluster is less regular than in a single galaxy, so careful modelling is necessary to understand the location of the critical lines and to derive the associated magnification. Some of the most distant galaxies known have been located by searching close to the critical lines of massive clusters where magnifications of 20–30× are typical (Ellis *et al.* 2001; Kneib *et al.* 2004); these systems would not have been detected without the boost in signal provided by gravitational lensing. As early galaxies are likely to be less massive and luminous than their later counterparts, this technique offers the only way to determine their abundance.

Not only do clusters magnify sources in their integrated brightness, rendering them more easily visible with our telescopes, but also lensing *enlarges the angular size* of a distant source, making it easier to determine its internal properties. The most distant galaxies are physically very small—about 10 times smaller than our Milky Way—and resolving them is a challenge for both the *Hubble Space Telescope* and large ground-based telescopes equipped with adaptive optics—a technique that corrects for atmospheric blurring. However, the combination of adaptive optics *and* gravitational magnification offers spectacular opportunities. A distant galaxy at a redshift of 3 is typically only 0.2–0.3 arcsec across, yet, when magnified by a factor of 30×, it is possible to secure spectroscopic data point- by-point across its enlarged image and to show that it has a rotating disc (Stark *et al.* 2008; figure 5*b*).

A tentative picture of early galaxy evolution is emerging from these and related studies. When the Universe was about 5 per cent of its current age, an abundant population of feeble low-mass galaxies formed from slowly cooling clumps of hydrogen gas. The energetic ultraviolet radiation from young stars in these early systems ionized the surrounding hydrogen gas. These early galaxies continued to assemble, both via mergers with one another and through continued accretion of hydrogen gas.

### (b) Weak lensing

**Figure 6.**
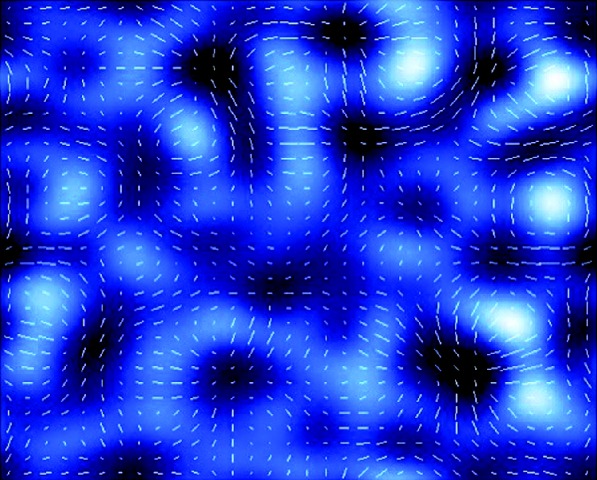
An idealized illustration of weak gravitational lensing. The blue image represents the projected mass distribution in a given area of the sky (white indicates a higher projected density of dark matter). The white tick marks represent the average shapes and orientations of a population of faint galaxies (assumed statistically to be round in shape) viewed through the dark matter. Where the dark matter is concentrated, the background galaxies are tangentially aligned around the structure; where the dark matter density is weak, the galaxies are aligned radially. The pattern of background galaxies can be used to infer the (invisible) distribution of foreground dark matter.

For those structures where the density in the lens lies below a critical value, multiple images cannot be formed. Yet, for most sight lines in the Universe, rays of light are frequently being deflected by dark structures. In this *weak lensing* regime, the principal signal is a small distortion in the shape of a background galaxy that depends on the *curvature* (or *second derivative*) of the foreground gravitational potential. In the idealized case, the observer sees the background source stretched (or sheared) tangentially around a circle whose centre is the lensing structure. In typical situations, the signal is too weak to be inferred for a single source. However, the presence of foreground structure can still be inferred by statistically analysing the distorted shapes of background galaxies in a given direction, assuming that, on average, galaxies are randomly oriented (figure 6).

Key aspects of the mathematics of weak lensing were developed soon after Einstein’s relativity theory was published, for example in early papers by Hermann Weyl and later in insightful articles by Gunn (1965, 1967). The first observational detection of a weak lensing signal was claimed by Tyson *et al.* (1990) in the field of a rich cluster. Techniques for robustly analysing the pattern of distortions of background galaxies and inverting these to map the foreground dark matter were developed by Kaiser and others (Kaiser 1992; Kaiser *et al.* 1995). Yet the clear detection of weak lensing from the large-scale distribution of dark matter along random sight lines, an effect known as ‘cosmic shear’, was not announced until 2000 (Bacon *et al.* 2000; van Waerbeke *et al.* 2000; Wittman *et al.* 2000). There are excellent reviews of this rapidly developing field by Bartelmann & Schneider (2001), Huterer (2002) and Refregier (2003).

#### (i) The distribution of dark matter on large scales

Weak gravitational lensing holds enormous promise in observational cosmology, as the technique, properly employed, can reveal the distribution of dark matter independently of any assumptions about its nature. However, the technical challenges are formidable. Foremost, the signal arising from the large-scale structure is small—amounting to a change in the ellipticity of a faint distant galaxy of only a few per cent. Secondly, as a statistical technique, a high surface density of measurable galaxies must be secured, so deep imaging is essential. Finally, as the Earth’s atmosphere smears the shapes of faint galaxies, painstaking corrections must be made to recover the cosmological signal. Ultimately, a space telescope with a panoramic imager may be required to realize the full potential.

**Figure 7.**
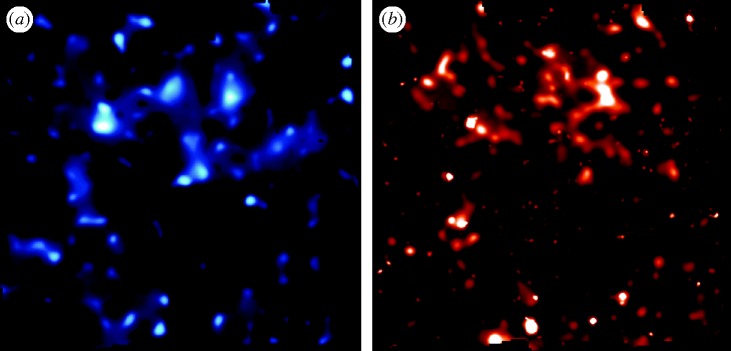
(*a*) Projected distribution of dark matter in the COSMOS field from the analysis of Massey *et al.* (2007*a*). The blue map reveals the density of dark matter as inferred from the pattern of weak distortions viewed in background galaxies by the *Hubble Space Telescope*. (*b*) Equivalent map for the baryonic matter as revealed by a combination of the stellar mass in galaxies imaged with the *Hubble Space Telescope* and hot gas imaged with the X-ray satellite *XMM–Newton*.

The early papers (cited above) analysed the strength of the signal to constrain the *amount* of dark matter per unit volume, confirming independently values from other methods. Later papers exploited the capabilities of the *Hubble Space Telescope* to produce the first projected map of its distribution (Massey *et al.* 2007*a*; figure 7*a*). This map of dark matter can be compared with that of the light in the same direction as revealed by visible galaxies and X-ray clusters (figure 7*b*). To first order, there is a reassuring similarity, indicative of the fact that dark matter acts as the gravitational framework (or *scaffolding*) for the normal baryonic material.

#### (ii) ‘Galaxy–galaxy lensing’: the extent of dark matter haloes around individual galaxies

In addition to tracing dark matter around clusters and on cosmic scales, a similar statistical technique can be applied around individual galaxies to detect their dark matter ‘haloes’. Suppose that we conduct a large spectroscopic survey of bright nearby galaxies and select a subset of systems of a particular class for which we have deeper imaging data. By ‘stacking’ the imaging data for that class of object, we can determine the average density of dark matter around a mean galaxy of this type to much larger radius than is possible using strong lensing (§3*a*(i)), and for a much wider variety of objects that may not be compact enough to act as strong lenses. Early detections of this so-called *galaxy–galaxy lensing*signal were made by Brainerd *et al.* (1996).

The Sloan Digital Sky Survey (York *et al.* 2000) is a good example of a recent imaging and spectroscopic survey that has been used to analyse this signal (Fischer *et al.* 2000; Sheldon *et al.* 2004). By correlating the positions of foreground galaxies of a given type with the effect that they have on the shapes of background galaxies, we can not only measure the extent of their dark matter haloes for given types, but also determine the extent to which such galaxies are, or are not, representative tracers of the underlying dark matter density field. Such analyses can be used to determine the shapes of the dark matter haloes (Parker *et al.* 2007) as well as to eliminate claimed non-Newtonian gravitational forces laws invoked to eliminate the need for dark matter (Tian *et al.* 2009). Finally, by comparing the dark matter haloes associated with galaxies as a function of their spatial positions in dense clusters, we can demonstrate that galaxies suffer violent environmental forces that work to strip their dark matter haloes (Natarajan *et al.* 2002, 2009).

Despite seemingly difficult technical obstacles only a few years ago, great strides have been made in using weak lensing to chart dark matter on large scales and around clusters and various galaxy populations. In the space of 3–4 years, we have confirmed the theoretical paradigm that emerged in the 1980s, which postulated that galaxies and clusters owe their existence to the gravitational clumping of a dominant dark matter density field. We can trace this dark matter around galaxies in statistically well-controlled samples and see how it differs in its extent and shape in various environments.

### (c) Microlensing

Einstein (1936) was not convinced that gravitational lensing would yield observable returns because the probability that two stars are sufficiently well aligned so that one magnifies the other is very low. But with panoramic imaging cameras, many tens of millions of stars can be monitored in the Milky Way. Together with the fact that there can be *relative motion* between the source and the lens, there is still the likelihood of observing an effect. Microlensing—a term introduced by Paczynski—generally refers to the case where either the source alone or both the source and the lens are unresolved. Consequently, the deflection and distortion of light from the background source cannot be seen. The key signature is a temporal brightening of the combined signal from the source plus lens as the one passes in front of the other. The time scale of the brightening can be anything from seconds to years, and the observed *light curve* gives information on the lens mass, the relative distances and the motion of the lensing object (assuming the background object is stationary). As microlensing is a transient phenomenon, an effective survey strategy is to monitor a dense star field repeatedly, searching for that rare occasion when an individual star increases its brightness. Of course, complicating such searches is the fact that many stars are genuinely variable in their output. Once a likely event has been triggered, it can be monitored more intensively to see if the light curve is of the form expected for microlensing. For a survey monitoring the dense star field in the centre of the Milky Way, the optical depth is about 2×10^−6^ (or an event at a particular time for every 400 000 stars being studied). In many cases, the light curves can be monitored to such exquisite precision that second-order effects, such as the motion of the Earth around the Sun, the finite size of the star that acts as a lens and even planets surrounding the lensing star, can be detected. With interferometric telescopes, shifts in the positions of the source may ultimately be detected.

Microlensing has had a major impact on astronomy in two areas, both involving monitoring of tens of millions of stars in the Milky Way or nearby Magellanic Clouds: (i) the search for dark matter in the Galactic halo in the form of compact objects of moderate mass (approx. 0.1 solar masses or less) and (ii) locating and assessing the abundance of extrasolar planets down to as low as 5 Earth masses.

#### (i) Dark matter searches in the Milky Way

**Figure 8.**
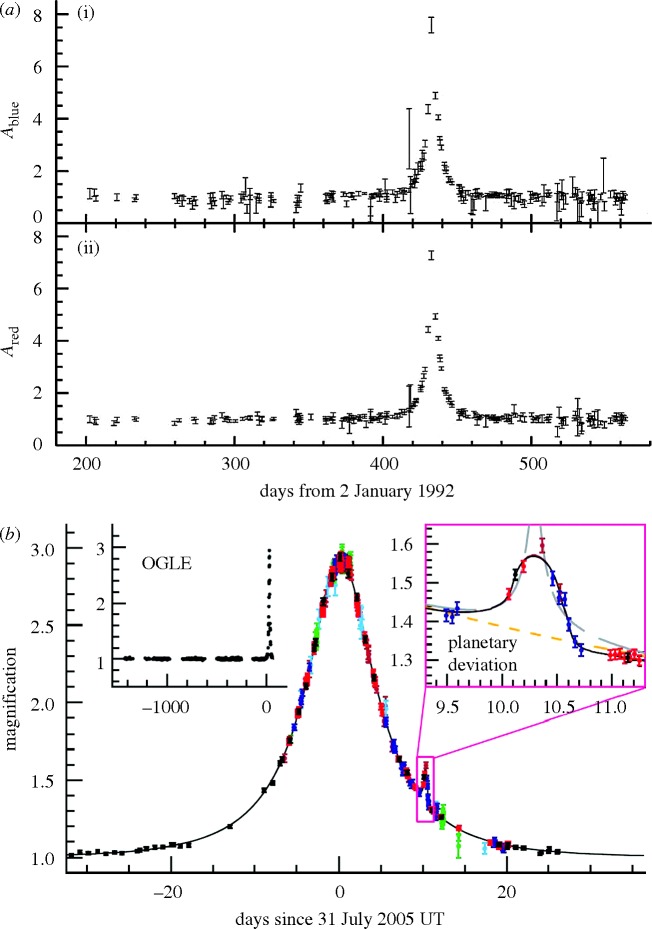
(*a*) Early halo microlensing event from the MACHO project (Alcock *et al.* 1993). The absence of a chromatic variation in the (i) blue and (ii) red light curves together with the duration of the event indicate lensing by an unseen line-of-sight compact object of approximately 0.1 solar masses. Too few such events have been detected for the dark matter in the Milky Way to be composed of such objects. (*b*) Detection of a low-mass exoplanet (OGLE 2005-BLG-390LB) via a small perturbation to the microlensing light curve (Beaulieu *et al.* 2006). Dataset sources: black, OGLE; green, Robonet; light blue, Canopus; red, Danish; dark blue, Perth; brown, MOA.

Paczynski (1986) first proposed using microlensing to test the hypothesis that unseen baryonic objects of substellar dimensions (generically known as massive compact halo objects, MACHOs) represented the source of the dark matter in the Milky Way. If this hypothesis could be rejected, the dark matter would most probably have to be non-baryonic, possibly in the form of a weakly interacting massive particle (WIMP) of mass 100 GeV to 10 TeV. Such microlensing events were first detected in 1993 by two teams (Alcock *et al.* 1993; Aubourg *et al.* 1993) undertaking a search for events in the Galactic halo by monitoring stars in the nearby Magellanic Clouds (figure 8*a*). Although many events were detected by these and successor surveys, the consensus that has emerged is that the Galactic dark matter is not dominated by dark compact sources in the mass range 10^−7^<*M*<15 in solar units (Tisserand *et al.* 2007). These surveys, completed largely in the 1990s, were arguably the first systematic use of gravitational lensing to make progress in cosmology since Eddington’s expedition.

#### (ii) Finding extrasolar planets

Despite not directly resolving the dark matter problem, the monitoring of stars in the centre of the Galaxy (where the optical depth is 20 times larger than in the direction of the Magellanic Clouds) has yielded almost a thousand possible events and gives a unique way to estimate the abundance of extrasolar planets. Again, the idea was first proposed by Paczynski.

In the case where the lens comprises a star with an orbiting planet, the light curve deviates from that expected for a single lens. Such a signal was first observed by Bond *et al.* (2004), leading to the detection of a 1.5 Jupiter mass planet. At the time of writing, at least 12 extrasolar planets have been detected by microlensing, with masses as low as approximately 5 Earth masses (figure 8*b*).

Microlensing has, like the other aspects of lensing reviewed above, hardly been thoroughly exploited. The gain of improved image quality and interferometric detections offer great promise, as I review in the final section.

## 4. The future: studies of dark energy and dark matter

We live in an exciting, if somewhat bewildering, time in terms of our understanding of the Universe. The concept of dark matter, as a significant component of the Universe, dates back to the 1970s following the realization that most galaxies are surrounded by unseen haloes. The null results of the Galactic microlensing surveys and the abundance of light elements produced in the big bang further suggest that dark matter is *non-baryonic* in form. Structure formation models can account for the presently observed large-scale distribution of galaxies if this non-baryonic matter is made of massive non-relativistic (i.e. ‘cold’) particles. Despite this progress, after 40 years, we have yet to detect the dark matter particle itself. Given how both strong and weak gravitational lensing have, in barely a decade, considerably added to our knowledge of the distribution and amount of dark matter, it is relevant to ask what these techniques can offer in the future.

Astronomical observations can define the spatial distribution of dark matter from small to large scales. Theory predicts this distribution in terms of the spectrum of physical scales over which the density of dark matter fluctuates. When input into numerical simulations, this *spectrum of density fluctuations* can grow with time to accurately reproduce the observed distribution of galaxies today. On large scales, weak lensing has demonstrated its ability to map dark matter fairly precisely, confirming the basic picture that dark matter forms the basis for structure formation.

However, on small scales, the standard ‘cold dark matter’ paradigm encounters some discrepancies with observations. Simulations predict that there should be many dwarf galaxies in attendance around large galaxies like our own Milky Way, but far too few are found. Whether this is a fundamental flaw in the dark matter picture can only be tested by searching for dark matter haloes directly (rather than counting visible dwarf galaxies, which represents an indirect test) (Kravtsov 2010).

This historical review has shown that the first technical breakthrough in gravitational lensing arose from *improved sensitivity* to faint lensed features—efficient detectors and large telescopes enabled us to see giant arcs and distant multiply imaged quasars. The second technical step forwards arose from *improved angular resolution*: *Hubble Space Telescope* images resolved the lenses in the SLACS survey, and improved ground-based imaging detected cosmic shear (weak lensing from large-scale structure).

The revolution in improved image quality continues. *Adaptive optics*—the use of rapidly adjustable optics to correct for atmospheric blurring by monitoring the incoming signal from a bright reference source—is now delivering images sharper than the *Hubble Space Telescope* on several large ground-based telescopes. A new generation of large optical/infrared telescopes (e.g. http://www.tmt.org) is being designed that will deliver higher-resolution data (Ellis in press). At radio wavelengths, where obscuration by interstellar and intergalactic dust is minimal, interferometers (http://www.skatelescope.org) are being planned to match these angular resolutions.

**Figure 9.**
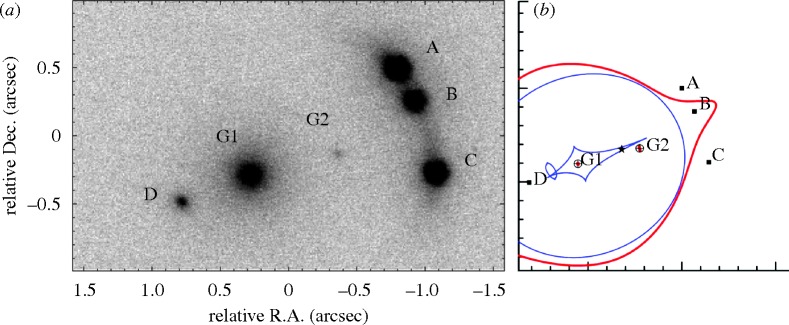
An illustration of milli-lensing in the multiply imaged B2045+265 quasar studied by McKean *et al.* (2007). (*a*) Near-infrared adaptive optics image taken with the Keck-II telescope. A–D are multiple images of the background quasar, and G1 is the primary lensing galaxy. A smooth lens model for G1 alone predicts image B to be the brightest image, yet it is anomalously fainter than images A and C. This is due to substructure in the lens arising from the low-mass satellite G2. (*b*) A lens model for the combined G1+G2 system matches the multiple image positions and fluxes. Caustics in the source plane are shown in blue and critical curves in the image plane in red.

This revolution in improved image quality promises enormous progress in charting the distribution of dark matter on fine scales and even, perhaps, constraining the nature of the dark matter particle. One of the most powerful probes will be *milli-lensing*—the detailed analysis of the positions and fluxes of multiply imaged sources seen at high redshift. When the light from a distant source is strongly lensed by a foreground galaxy, positional errors and anomalous fluxes of the images (compared with the predictions of a fiducial model) contain valuable information on the distribution of very low-mass (less than 10^9^ in solar units) haloes close to the lensing galaxy (Metcalf & Madau 2001; McKean *et al.* 2007; figure 9). Moreover, fine structure is similarly induced in extended lensed images such as arcs located in lensing clusters. Such probes of the fine-scale distribution of dark matter will require exquisite angular resolution, at either near-infrared or radio wavelengths, and the extensive monitoring of hundreds of lensed sources. At present, the number of well-modelled lens systems is too few. The first step will be to undertake comprehensive imaging surveys to find much larger samples. Some of these surveys are already under way.

If the puzzle of resolving the dark matter question were not sufficiently embarrassing for astronomers, consider the discovery from two studies of distant supernovae in the 1990s (Riess *et al.* 1998; Perlmutter *et al.* 1999) that the cosmic expansion is not slowing down, as expected in a Universe dominated by gravitating matter (dark and visible), but actually accelerating! This result, confirmed independently with improved precision in subsequent surveys (e.g. Astier *et al.* 2006), implies the presence of an energy density with a negative relativistic pressure that opposes the attractive properties of gravity.

The moniker *dark energy* was invented largely to hide our ignorance of this property of space. An explanation that is gaining much momentum at present is that dark energy may even be an illusion arising from an incomplete description of Einstein’s gravity. Physicists were reluctant to abandon Newtonian physics at the beginning of the twentieth century and so might some be for general relativity today. Either way, the resolution of the dark energy problem offers the prospect of an exciting revision of our understanding of the Universe.

As is often the case when there are few observations, theories for dark energy abound! Weak gravitational lensing offers possibly the best prospect for constraining our untamed theorists and tracking the detailed properties of dark energy. Since the putative energy opposes gravity, it inhibits the growth of structure: thus a measure of the growth of the spectrum of dark matter density fluctuations with cosmic time encapsulates the competition between dark energy and gravity and enables us to directly track what dark energy might be.

The dark matter maps presented earlier are projected two-dimensional versions and so the challenge will be to dissect these into three-dimensional versions that contain time-dependent information on the growth of structure. Deep multi-colour imaging of the background galaxies can aid in this respect, enabling us to slice the dark matter maps of the Universe in cosmic time. Early results (Massey *et al.* 2007*b*) have demonstrated that such weak lensing *tomography* is feasible, but so far they have only been conducted in areas of sky that are far too small for robust results. Again, the challenge is a technical one—building a facility that can not only achieve the imaging acuity to detect the small weak lensing distortion but also scan huge areas of sky to ensure statistically significant results.

The Large Synoptic Survey Telescope (http://www.lsst.org/lsst) is a proposed ground-based facility that would certainly tackle this challenge. However, ultimately, many believe that a space observatory such as the proposed NASA–DoE *Joint Dark Energy Mission* (http://jdem.gsfc.nasa.gov) or ESA’s *Euclid* mission (http://sci.esa.int/science-e/www/area/index.cfm?fareaid=102) will be required to fully realize the potential of gravitational lensing, at least at optical wavelengths.

In conclusion, it is interesting to speculate what Einstein would think of the progress summarized in this review in the light of his 1936 remark ‘of course there is no hope of observing this phenomenon’. Fortunately, there are sufficient examples of Einstein changing his mind (the predicted deflection, the expansion of the Universe and the presence of the cosmological constant) that I am confident he would be as enthusiastic about its applications as the rest of the present-day astronomical community!

## Footnotes

1 Sloan Lens ACS Survey.
